# Interleukin17A Promotes Postoperative Cognitive Dysfunction by Triggering β-Amyloid Accumulation via the Transforming Growth Factor-β (TGFβ)/Smad Signaling Pathway

**DOI:** 10.1371/journal.pone.0141596

**Published:** 2015-10-28

**Authors:** Ayong Tian, Hong Ma, Rongwei Zhang, Wenfei Tan, Xiaolong Wang, Binyang Wu, Jun Wang, Chengfu Wan

**Affiliations:** 1 Department of Anesthesiology, the first Affiliated Hospital of China Medical University, Nanjing North Street 155, Shenyang, Liaoning, China; 2 Department of Gerontology and Geriatrics, the first Affiliated Hospital of China Medical University, Nanjing North Street 155, Shenyang, Liaoning, China; 3 Department of Neurology, the first Affiliated Hospital of China Medical University, Nanjing North Street 155, Shenyang, Liaoning, China; 4 Department of Pain Medicine, the first Affiliated Hospital of China Medical University, Nanjing North Street 155, Shenyang, Liaoning, China; Texas Tech University Health Science Centers, UNITED STATES

## Abstract

Although postoperative cognitive dysfunction (POCD) is relatively common in elderly patients who have undergone major surgery, the mechanisms underlying this postoperative complication are unclear. Previously, we have investigated the role of cytokine-mediated hippocampal inflammation in the development of POCD in a rat model. Here, we sought to determine in mice the role of cytokine interleukin17A (IL17A) in POCD and to characterize the associated signaling pathways. Old mice underwent hepatectomy surgery in the presence or absence of IL17A monoclonal antibody, and cognitive function, hippocampal neuroinflammation, and pathologic markers of Alzheimer’s disease (AD) were assessed. We found that the level of IL17A in the hippocampus was increased in hepatectomy mice and that cognitive impairment after surgery was associated with the appearance of certain pathological hallmarks of AD: activation of astrocytes, β-amyloid_1-42_ (Aβ_1–42_) production, upregulation of transforming growth factor-β (TGFβ), and increased phosphorylation of signaling mother against decapentaplegic peptide 3 (Smad3) protein in the hippocampus. Surgery-induced changes in cognitive dysfunction and changes in Aβ_1–42_ and TGFβ/Smad signaling were prevented by the administration of IL17A monoclonal antibody. In addition, IL17A-stimulated TGFβ/Smad activation and Aβ_1–42_ expression were reversed by IL17A receptor small interfering RNA and a TGFβ receptor inhibitor in cultured astrocytes. Our findings suggest that surgery can provoke IL17A-related hippocampal damage, as characterized by activation of astrocytes and TGFβ/Smad pathway dependent Aβ_1–42_ accumulation in old subjects. These changes likely contribute to the cognitive decline seen in POCD.

## Introduction

Postoperative cognitive dysfunction (POCD) is a subtle disorder of perception, memory, and information processing after surgery [1]. A multicenter study has shown that POCD was present in 26% and 10% of patients at 1 week and 3 months, respectively, after surgery [2]. POCD not only has a significant impact on patient health, but it has also been associated with greater morbidity and delays in functional recovery [3]. For these reasons, further research regarding the molecular mechanisms and pathways involved is critical for better understanding and managing POCD.

Although it was previous suggested that the type of anesthesia may contribute to the development of POCD, a study on the relationship between POCD and anesthesia showed that the incidence of POCD was similar after regional and general anesthesia, indicating that anesthesia does not appear to contribute to the development of POCD [4]. More recently, research efforts have focused on the role of the type of surgical intervention. Our laboratory and others have shown that hippocampal inflammation, as demonstrated by a local increase in the transcription and expression of cytokines in aged mice, increased following peripheral surgery [5, 6, 7, 8]. In Alzheimer’s disease (AD), hippocampal inflammation occurs in tandem with the accumulation of β-amyloid (Aβ), and this process is believed to be central to the pathogenesis of AD [9]. Cytokine levels are elevated in AD patients relative to controls [10], and astrocyte activation was linked to the deposition of amyloid plaques in the AD brain [11]. Given these similarities, the molecular mechanisms underlying POCD and AD may overlap.

TGFβ, a cytokine overexpressed in the brain of AD patients [12], was shown to induce APP synthesis *in vivo* and *in vitro* and to promote Aβ formation by a transcriptional mechanism involving the signaling mother against decapentaplegic peptide 3 (Smad3) signaling pathway in astrocytes [13]. The TGFβ/Smad signaling pathway is believed to be involved in a wide spectrum of physiological and pathological mechanisms that are implicated in several neurological disorders, including Parkinson's disease [14], multiple sclerosis [15], and AD [16]. To date, there is no direct evidence linking abnormal regulation of the TGFβ/Smad signaling pathway to POCD.

Here, we describe a new role for cytokine IL17A, a cytokine produced by Th17 cells, that plays key roles in tissue inflammation and autoimmune responses [17, 18]. IL-17 has become an important target for treating different forms of inflammatory disorders, and many studies have demonstrated that increased IL-17 expression is associated with inflammatory diseases in either human patients or animal disease models [19, 20, 21]. IL-17 also plays critical roles in the pathogenesis of some inflammation-related diseases in the central nervous system [22, 23, 24]. The aims of this study are to determine whether IL17A-triggered hippocampal inflammation accompanied by pathologic markers of AD can be observed following hepatectomy in mice and to determine which signaling pathway contributed to these biochemical changes.

## Materials and Methods

### Reagents

IL17A monoclonal antibody was purchased from R&D Systems (Minneapolis, MN, USA), and rat anti mouse IgG2a was from Abcam (Cambridge, UK). Antibodies for IL17A, IL6, TGFβ, GFAP, APP, and Aβ_1–42_ (Western blot) were purchased from Cell Signaling Technology (Beverly, MA, USA). Antibodies for Smad2/3 and pSmad2/3 (Ser 423/425, Western-blot) were purchased from Upstate Biotechnology (Billerica, MA, USA), and antibodies for pSmad2/3 (Immunofluorescence), GFAP, Aβ_1–42_ (immunohistochemistry) were from Santa Cruz Biotechnology (Santa Cruz, CA, USA). The pharmacological inhibitors for TGFβ receptorI/II (TβRI/II, LY2109761) were purchased from Selleck Chemicals (Houston, TX, USA), and IL17RA siRNA was from Wanlei life sciences (Shenyang, China).

### Animals and Surgical Procedures

C57BL/6 14-mo-old male mice were housed in polypropylene cages and maintained at 23°C under a 12-h light/dark cycle in a humidity controlled environment with food and water available *ad libitum*. Experiments were approved by the China Medical University Animal Care and Use Committee and adhered to the guidelines for care and use of laboratory animals from the Chinese Academy of Science.

Mice were trained for five consecutive days on the Morris water maze (MWM) test and for 3 days on the T maze test, then randomly assigned to one of three groups: 1) Control group (group C; n = 48), mice received no intervention; 2) Anesthesia group (groupA; n = 48), mice received general anesthesia with 50mg/Kg sodium pentobarbital injected intraperitoneally (i.p.); And 3) Anesthesia-plus-surgery group (group SA; n = 48), mice underwent partial hepatectomy under general anesthesia. For hepatectomy, the liver was exposed through a 1–2 cm midline abdominal incision. The left lateral lobes of the liver (30% of the organ) were excised. The wound was then infiltrated with 0.25% bupivacaine and closed by sterile suture. For the IL17A blockade test, there were four groups: 1) Control group (group C; n = 16), mice received no intervention; 2) Surgery group (group SA; n = 16), mice only underwent partial hepatectomy under general anesthesia; 3) Surgery plus IL17A monoclonal antibody group (group SI; n = 16), mice were treated as in the SA group plus administration of IL17A monoclonal antibody during the surgery; and 4) Surgery plus IgG2a control group (group SC, n = 16), mice were treated as in the SI group but rat anti mouse IgG2a was administered in place of IL17A monoclonal antibody.

### Intracerebroventricular Cannulation and Experimental Protocol

Mice were prepared with an intracerebroventricular (ICV) cannula, as previously described [25]. In brief, mice were anesthetized with an i.p. injection of sodium pentobarbital (50mg/Kg) and positioned in a stereotaxic instrument (NDY-X, Tianjin, China) so that the plane formed by the frontal and parietal bones was parallel to horizontal zero. A stainless steel cannula was placed in the left lateral cerebral ventricle at the the following coordinates: 1.6 mm lateral and 1.0 mm anteroposterior to bregma and 2.0 mm horizontal from the dura mater). Mice recovered for at least five d prior to surgery. IL17A monoclonal antibody was dissolved in sterile saline and 5 μl (4 μg) of rat anti mouse IgG2a or IL-17A antibody 5 μl (4 μg) was injected during surgery over a 30 sec period using a syringe pump.

### Cognitive Test: MWM

The MWM is a hippocampal-related test of spatial learning for rodents [26]. A circular, black painted pool (diameter, 180 cm; depth, 50 cm) was filled with water to 30 cm. An invisible platform (diameter, 10 cm) was submerged 1 cm below the water surface in one quadrant. Mice were tested daily for 5 consecutive days, three trials per day, before surgery and 7 days after surgery. For each trial, the animal was placed in a different quadrant facing the pool wall and allowed to swim and self discover the hidden platform. Mice who failed to locate the platform within 120 s were guided gently to the platform. A minimum interval of 10 min was allowed between each trial. Animals underwent surgery on day 6. Swimming distance, speed, and escape latency to the platform were recorded by a video camera mounted to the ceiling, and digital images were analyzed by water maze software (HVS image, Buckingham, UK). Additionally, on postoperative days 1, 3, and 7, mice were subjected to a probe test in which the platform was removed and the mouse was allowed to swim for 90 sec. The time spent in the quadrant previously containing the submerged platform was recorded and represented as an index of memory. Mice were sacrificed on postoperative days 1, 3, and 7 (n = 16 per day) after the cognitive test.

Hippocampal tissues from mice in each group were rapidly dissected and stored at −70°C until they were used for messenger RNA (mRNA) and Western blot studies. Remaining animals in each group were sacrificed and transcardially perfused with heparinized saline followed by 300 ml of ice-cold 4% paraformaldehyde in PBS (pH 7.4). Brains were dissected and post-fixed in 4% paraformaldehyde in PBS for 4 h and subsequently embedded in paraffin and sectioned for histopathological studies.

### Cognitive Test: T maze

The T-maze, which has been used in a variety of ways to assess the cognitive ability of an animal [27], was made of black plexiglass and composed of a start arm (length, 70 cm; width, 20 cm; height, 20 cm) and two identical goal arms (length, 70 cm; width, 20 cm; height, 20 cm) with a sugar plate located 5 cm from the end of each goal arm. Mice were maintained on a restricted feeding schedule at 85% of their free-feeding weight. They were habituated to the maze and were accustomed to reward foods (a small sugar). Each trial consisted of a sample-run and a choice-run. On the sample-run, mice were forced to enter either left or right arm to get sugar, while the other arm was blocked by a slide door. On the choice-run, the blocked door was removed and the mice were allowed to choose either arm freely. The interval time between the sample- and the choice- run was 10 sec. If mice entered the previously unvisited arm, the mice were rewarded. The delayed alternation was then prolonged to 1 and 3 min. Each daily session consisted of 5 trials, and mice were run one trial at a time with an intertribal interval of 10 min.

### Primary astrocyte cultures

Primary astrocyte cultures from wild-type C57BL/6 mice were established from neonatal cerebra, as described previously [28]. Cells were cultured in DMEM (high glucose, 6 g/L), 2 mM glutamine, 0.1 mM nonessential amino acid mixture, 0.1% gentamicin, and 10% FBS (HyClone, Logan, UT, USA). After 2 weeks in culture, oligodendrocytes and microglia were separated from astrocytes by mechanical dislodgment. The astrocyte cultures were monitored for purity by immunofluorescent staining for an astrocytic marker, GFAP. Routinely, > 97% of cells in the cultures were positive for GFAP.

### Quantitative Real-Time Polymerase Chain Reaction

Total RNA was extracted from tissue specimens using Trizol Reagent (Invitrogen, Carlsbad, CA, USA). cDNA was synthesized with the QuantiTect Reverse Transcription Kit with integrated removal of genomic DNA (Qiagen, Valencia, CA, USA) according to the manufacturer’s protocol. Quantitative real time PCR was performed using an ABI 7500 PCR system, and levels of mRNA for IL17A, IL6, TGFβ, Smad2, Smad3, and APP from all groups were assayed according to the manufacturer's protocols. The amplification conditions were 8 min at 95°C, followed by 45 cycles of 95°C for 5 s, 60°C for 34 s, and 72°C for 15 s. Specific primers were designed using Primer5 (Table 1). For normalization of total RNA in each sample, β-actin gene was used as an endogenous control, and all values were expressed relative to the expression of β-actin (2^-ΔΔct^).

**Table 1:** Specific primer sequence for real-time PCR.

Gene	Forward (5’-3’)	Reverse (5’-3’)
IL17A	CTGTGTCTCTGATGCTGTTGC	GTGGAACGGTTGAGGTAGTCT
IL6	ACTTCCATCCAGTTGCCTTCTT	TCATTTCCACGATTTCCCAGA
TGFβ	GCAACAATTCCTGGCGTTACCT	GAAAGCCCTGTATTCCGTCTCC
Smad2	TATCGGAGGCAGACAGTAACAAGTA	GAAACAGATTCCACAAGGTGCTTTA
Smad3	TTCTGCCGCTTTCGTATTTATGTA	TACTCTAATGCTTGTGGATCTTGA
APP	GAATGGAAAGTGGGAGTCAGA	CAACTAGGCAACGGTAAGGAA
β-actin	CTGTGCCCATCTACGAGGGCTAT	TTTGATGTCACGCACGATTTCC

### Western Blot Analysis

Hippocampal tissue samples were homogenized in ice-cold lysis buffer containing protease inhibitors and centrifuged at 12,000 *g* for 10 min at 4°C. The supernatant was collected, and the protein concentration was determined by the bicinchoninic acid (BCA, Sigma, St. Louis, MO, USA) method using bovine serum albumin as the standard. Protein samples were resolved by 10% sodium dodecyl sulfate-polyacrylamide gel electrophoresis (SDS-PAGE), and transferred onto polyvinylidenedifluoride (PVDF) membrane (Millipore, Billerica, MA, USA). Membranes were blocked in 5% skim milk and incubated overnight at 4°C with primary antibodies against IL17A (1:100), IL6 (1:100), TGFβ (1:100), GFAP (1:100), APP (1:100), Aβ_1–42_ (1:100), Smad2/3 (1:100) and pSmad2/3 (1:100). Membranes were incubated with HRP-labeled secondary antibodies (1:5000), and proteins were detected by enhanced chemiluminescence (ECL; Amershan Biosciences, Chalfont St. Giles, UK) according to the manufacturer’s instructions. Signals were quantitatively analyzed with Quantity-One software (Bio-Rad). B-actin was used as a protein-loading control.

### Immunohistochemistry

Sections (5 μm) were cut from paraffin embedded hippocampal tissue samples for immunohistochemistry. In brief, sections were incubated in 0.3% hydrogen peroxide-methanol solution, blocked in 5% bovine serum albumin, and incubated overnight at room temperature with rabbit polyclonal anti- GFAP (1:100), rabbit polyclonal anti- Aβ antibody (1:100), or rat immunoglobulin IgG (1:100) as the negative control. After washing with PBS, biotin-conjugated secondary antibodies were applied (1:100) at 37°C for 30 min. Sections were washed with PBS and incubated with diaminobenzidine (DAB) for detection. Finally, the sections were mounted on gelatin-coated slides, dehydrated in an ascending alcohol series, cleared in xylene, and photographed under bright field microscopy (magnification, ×400). For quantification, the positive cells in the hippocampus were estimated by using mean integrated optical density (OD). MetaMorph software system (Sunnyvale, CA, USA) was used for image analysis.

### Double immunofluorescene labeling assay

Cells were fixed in 4% paraformaldehyde for 15 min and washed in PBS. After cells were blocked with normal rabbit serum for one h at room temperature, they were incubated with rabbit anti pSmad2/3 (1:50) and rat anti mouse GFAP (1:50) at 4°C overnight. Following washes, cells were incubated with secondary antibodies containing a mixture of Cy3-conjugated goat anti rabbit IgG (1:100) and fluorescein isothiocyanate (FITC)-conjugated goat anti rat IgG (1:100) at room temperature for one h. Cell nuclei were counterstained with 4',6-diamidine-2'- phenylindoledihydrochloride (DAPI, Sigma) at room temperature for five min. The cells were then examined under fluorescence with the Nikon Eclipse E800 microscope (Tokyo, Japan) and the Spot advanced digital camera (Diagnostic Instruments, Sterling Heights, MI, USA). Fluorescence images were merged using Andor IQ software (Andor Technology, Belfast, Northern Ireland).

### Statistical Analysis

All data are presented as mean±SEM. Statistical analysis was performed with analysis of variance (ANOVA) followed by the Student-Newman-Keuls multiple comparison test for numerical data. Unpaired Student t test was used for comparisons between two groups. *P* < 0.05 was considered significant.

## Results

### Partial hepatectomy induced spatial memory impairment

**Fig 1:**
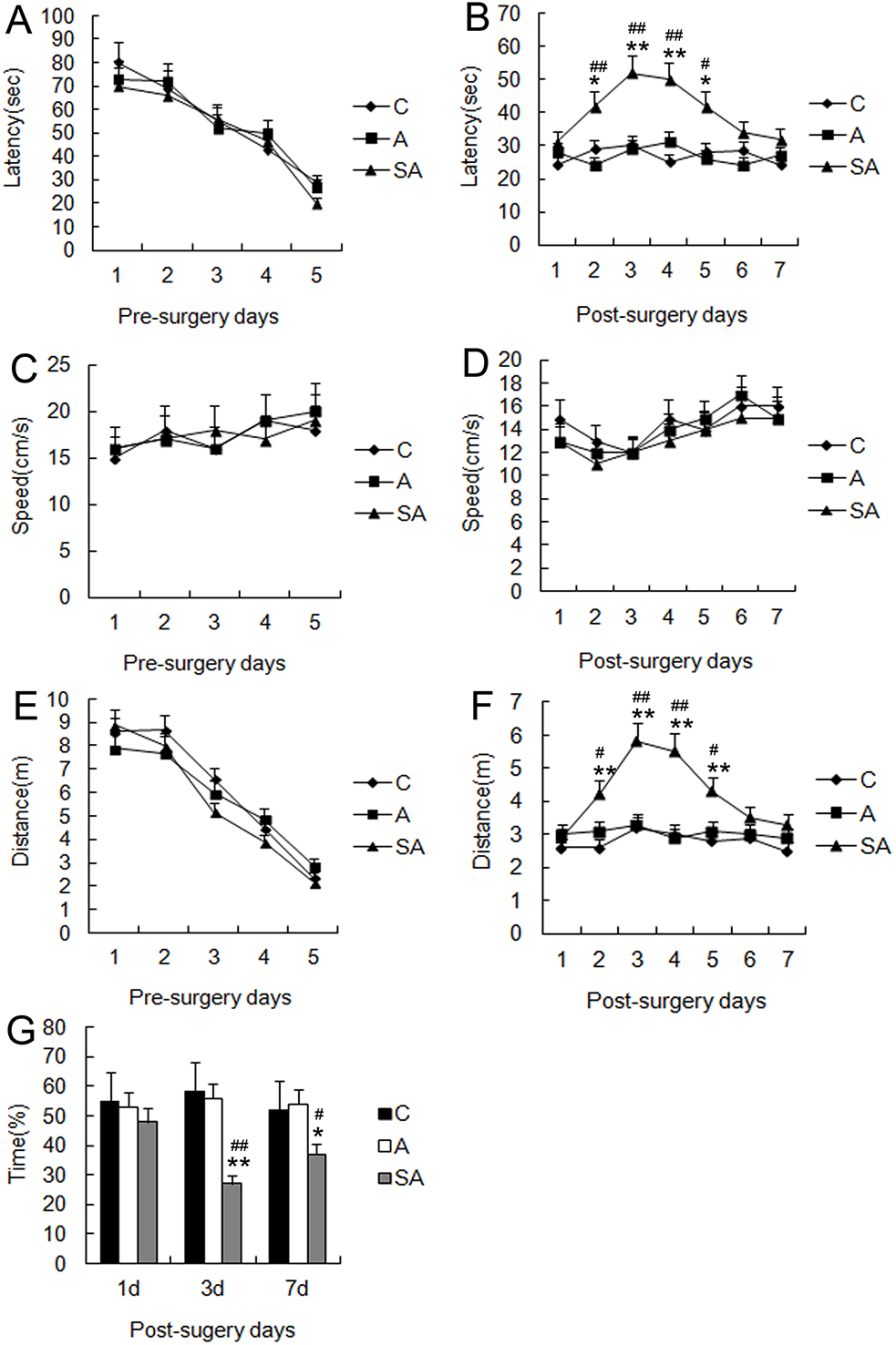
Performance in the hidden-platform Water Maze Test by hepatectomy surgery mice. The Morris water maze (MWM) was used to train mice for 5 days before surgery and to test memory formation changes for 7 days after surgery. (A) Latency to find the platform across testing days. (B) Latency to find the platform after surgery. (C) Swim speed across testing days. (D) Swim speed after surgery. (E) Distance to platform across testing days. (F) Distance to platform after surgery. (G) The probe test in the Morris water maze. Data are represented as means ± SEM. *P<0.05; **P<0.01 vs. C group at the corresponding time point. #*P*<0.05; ##*P*<0.01 vs. A group at the corresponding time point. Groups were as follows: C, control; A, anesthesia; SA, surgery plus anesthesia (n = 16).

**Fig 2:**
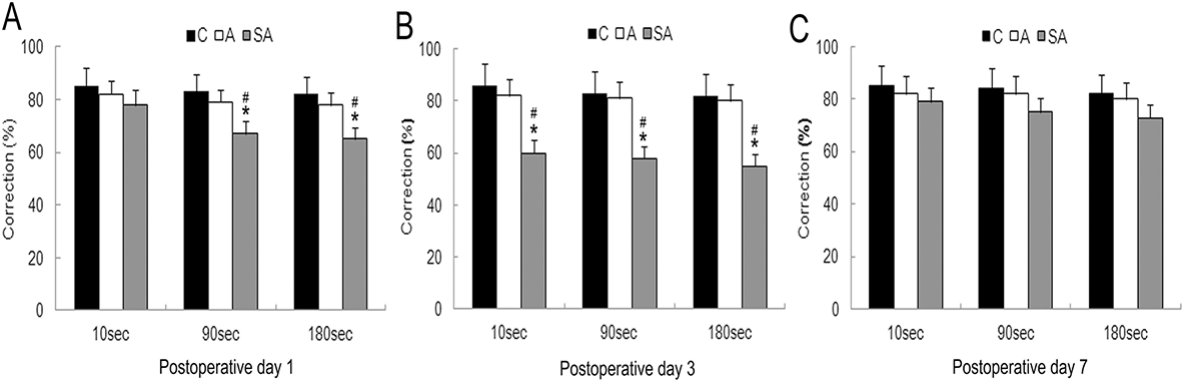
Impairment of spatial working memory on T maze test in hepatectomy surgery mice. Spatial working memory was examined using a T maze and was tested using various intervals at the 10 sec, 1 min, and 3 min delay on postoperative day 1 (A), 3 (B) and 7 (C). Data are represented as means ± SEM. *P<0.05; vs. C group at the corresponding time point. #*P*<0.05; vs. A group at the corresponding time point. Groups were as follows: C, control; A, anesthesia; SA, surgery plus anesthesia (n = 8).

To elucidate the effect of partial hepatectomy on learning and memory, we conducted the MWM test, a highly sensitive and well validated test of cognitive function for rodents. It is particularly sensitive at testing hippocampus-dependent learning and memory formation. In our tests, mice in all groups were able to find the platform, and the escape latency gradually decreased over the five consecutive training days. By the fifth training day, the latency was reduced to 51%, 46%, and 50% of the first day of training in control group (group C), anesthesia group (group A), and anesthesia-plus-surgery group (group SA), respectively (*P*>0.05, Fig 1A). On postoperative day 1, mice in all three groups were able to locate the platform within a short time (24–31 sec). However, compared with group C, mice in group SA exhibited a significant increase in escape latency time from postoperative day 2 (42 sec) to postoperative day 4 (50 sec), with a peak latency time on postoperative day 3 (*P*<0.01, Fig 1B). The latency times in group SA group were significantly greater than group A at the corresponding time points (day 3: *P*<0.01; day five: *P*<0.05, Fig 1B). Swimming speed was not significantly different among groups at any corresponding time points (*P*>0.05, Fig 1C and 1D), and the pattern of change in swimming distance was the same as that of latency (*P*<0.05 or 0.01, respectively; Fig 1E and 1F). Our probe tests revealed that the time spent in the quadrant with the previously located hidden platform was significantly less in group SA (44, 27, and 37%) relative to group A (53, 54, and 56%) on postoperative days 1, 3, and 7, respectively (day 3: *P*<0.01; day 7: *P*<0.05, Fig 1G). Overall, these results indicated that surgery itself contributed to reduced cognitive function in POCD. We also tested spatial working memory function in all groups by using the T-maze non-matching-to-place task. In this task, the spatial response of mice must change from trial to trial according to the sample information given immediately before each choice. Normal mice readily alternate their responses on two-choice mazes, resulting in good non-matching to place, but damage to the septo-hippocampal system profoundly impairs this response. In the present research, control mice showed excellent non-matching to place performance for a sugar during the training on postoperative day 1, 3, and 7. In contrast, the surgery mice were significantly impaired and exhibited worse performance when the interval between sample-run and test-run was delayed 1 and 3 min on postoperative day 1 and 3 (*P*<0.05, Fig 2A–2C). There was no statistically significant difference between anesthesia group and control group (*P*>0.05).

### Partial hepatectomy induced IL17A-related cytokines expression, astrocytes activation, APP and Aβ_1–42_ production, and Smad2/3 protein phosphorylation in the hippocampus

**Fig 3:**
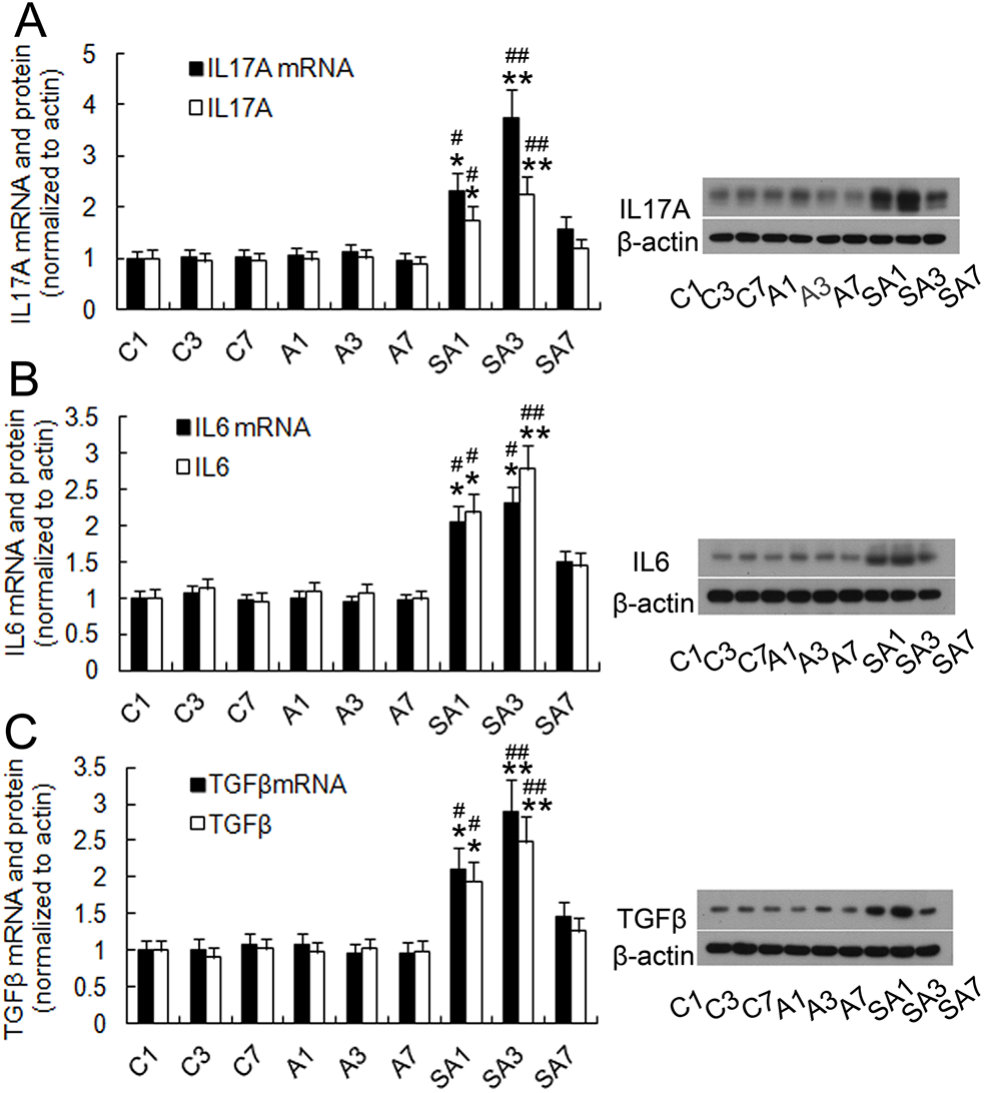
Expression of IL17A and differentiation-related cytokines in the hippocampus following hepatectomy in mice. The mRNA and protein expression of IL17A, IL6, and TGFβ in hippocampus were measured by qRT-PCR and western blotting assay on postoperative days 1, 3, and 7. Surgery resulted in increased hippocampus mRNA and protein of IL17A (A), IL6 (B), and TGFβ (C) relative to mice receiving anesthesia alone or control mice. Data are represented as means ± SEM. *P<0.05; **P<0.01 vs. C group at the corresponding time point. #*P*<0.05; ##*P*<0.01 vs. A group at the corresponding time point. Groups were as follows: C, control; A, anesthesia; SA, surgery plus anesthesia (n = 8).

**Fig 4:**
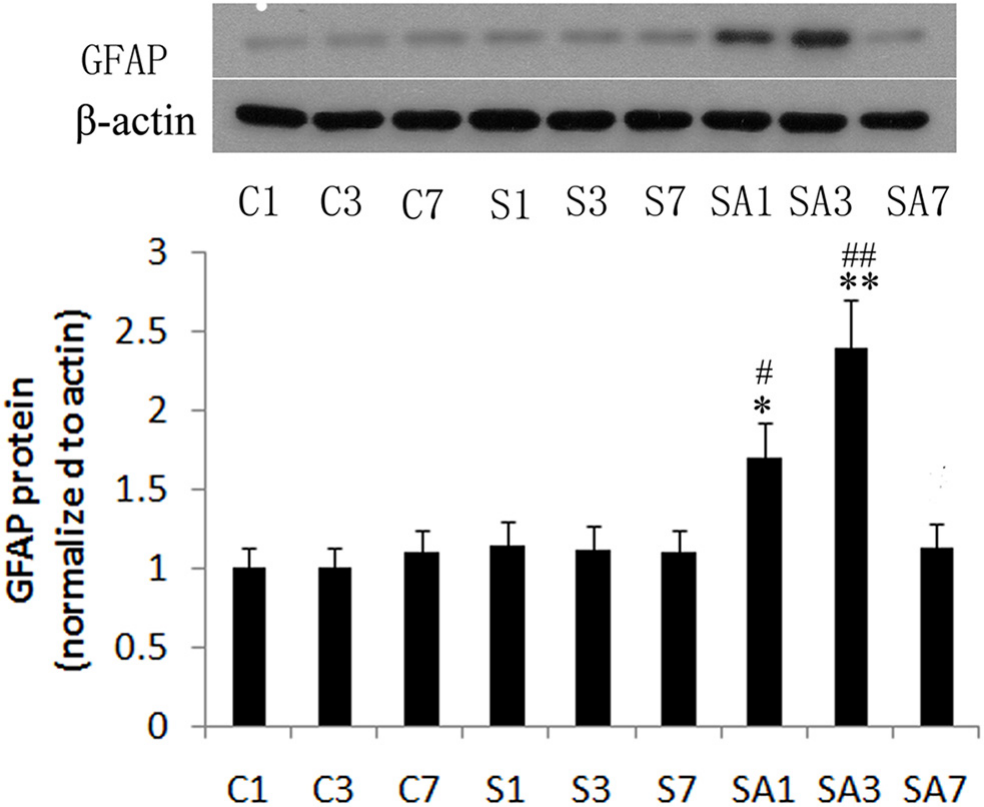
Glial fibrillary acidic protein (GFAP) expression in the hippocampus following hepatectomy in mice. Representative bands of the hippocampus illustrating expression of GFAP from control group at 1 day (C1), 3 days (C3), and 7 days (C7); from anesthesia group at 1 day (A1), 3 days (A3), and 7 days (A7); or from surgery plus anesthesia group at 1 day (SA1), 3 days (SA3), and 7 days (SA7). Data are represented as the mean ± SEM. *P<0.05; **P<0.01 vs. C group at the corresponding time point. #*P*<0.05; ##*P*<0.01 vs. A group at the corresponding time point. Groups were as follows: C, control; A, anesthesia; SA, surgery plus anesthesia (n = 8).

**Fig 5:**
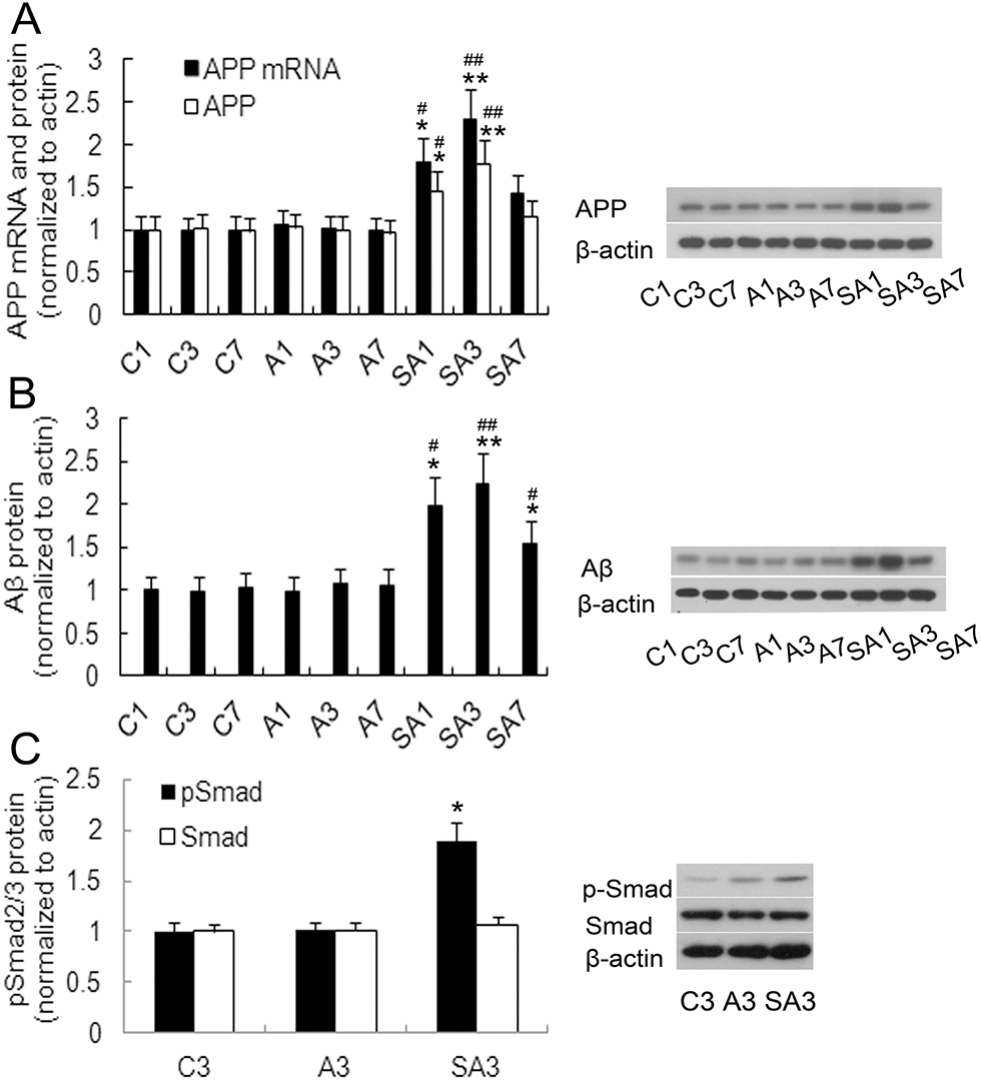
Expression of APP, Aβ_1–42_ protein, and phospho-Smad protein in the hippocampus following hepatectomy in mice. The mRNA and protein measurements of APP **(**A) and Aβ_1–42_ (B) protein in hippocampus were made at 1 day (C1), 3 days (C3), and 7 days (C7) from control group; at 1 day (A1), 3 days (A3), and 7 days (A7) from anesthesia group or at 1 day (SA1), 3 days (SA3), and 7 days (SA7) from surgery plus anesthesia group. (C) phospho-Smad protein (pSmad) was detected by western blotting assay on postoperative day 3. Data are represented as means ± SEM. *P<0.05; **P<0.01 vs. C group at the corresponding time point. #*P*<0.05; ##*P*<0.01 vs. A group at the corresponding time point. Groups were as follows: C, control; A, anesthesia; SA, surgery plus anesthesia (n = 8).

**Fig 6:**
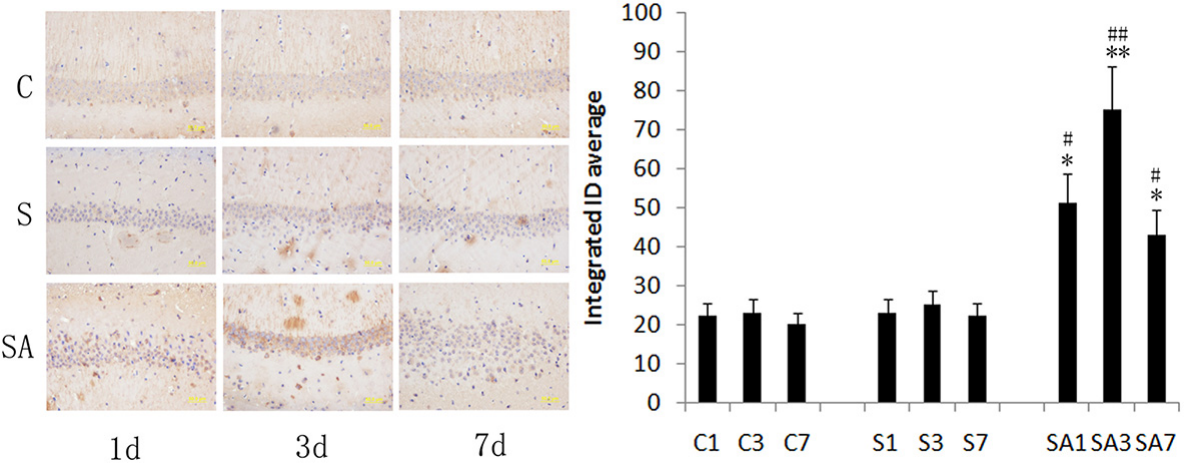
Aβ_1–42_ protein expression detected by immunohistochemistry in the hippocampus following hepatectomy in mice. Representative photomicrographs of immunostaining of the hippocampus for expression of Aβ_1–42_ from the control group at days 1 (C1, A), 3 (C3, B), and 7 (C7, C); the anesthesia group at days 1 (A1, D), 3 (A3, E), and 7 (A7, F); and the surgery plus anesthesia group at days 1 (SA1, G), 3 (SA3, H), and 7 (SA7, I) (magnification ×400). (J) Histogram showing the integrated OD average in the three groups. Data are represented as the mean ± SEM. *P<0.05; **P<0.01 vs. C group at the corresponding time point. #*P*<0.05; ##*P*<0.01 vs. A group at the corresponding time point. Groups were as follows: C, control; A, anesthesia; SA, surgery plus anesthesia (n = 8).

To determine if surgery induced IL17A-related cytokine production, hippocampal IL17A, IL6, and TGFβ mRNA levels were measured on postoperative days 1, 3, and 7. IL6 and TGFβ play critical roles in the development of the Th17 response and are positive regulators in the differentiation of Th17 cells. Anesthetics did not significantly increase IL17A, IL6, and TGFβ mRNA expression at any time point relative to controls (*P*>0.05). However, on postoperative day 1 and 3, the levels of IL17A, IL6, and TGFβ mRNA expression were elevated in group SA, peaking on day postoperative 3 (*P*<0.01). Likewise, protein expression of IL17A, IL6, and TGFβ was also markedly elevated on postoperative days 1 and 3 (day 1: *P*<0.05; day 3: *P*<0.01, Fig 3A–3C). GFAP is an often employed marker of astrocytic activation, an event which is thought to occur early during the formation of amyloid plaques [29]. Therefore, we investigated GFAP expression and found that hippocampal GFAP expression significantly increased on postoperative days 1 and 3 in group SA (day 1: *P*<0.05; day 3: *P*<0.01, Fig 4) and improved by postoperative day 7. We next measured APP and Aβ_1–42_ levels in order to investigate the effect of surgery on the production of Aβ. Hepatectomy was associated with an increase of APP and Aβ_1–42_ levels on postoperative day 1 and 3 (day 1: *P*<0.05; day 3: *P*<0.01, Fig 5A and 5B) and Aβ_1–42_ level up to postoperative day 7. The level on day seven was significantly higher than group C and group A (*P*<0.05). Immunohistochemistry was also used to detect Aβ_1–42_ expression, and similar results were obtained (Fig 6). Additionally, hepatectomy was associated with a significant increase in p Smad2/3 protein levels in the hippocampus relative to group C or group A on postoperative day 3 (*P*< 0.05, Fig 5C).

### Partial hepatectomy induced hippocampus pathological injury could be rescued by IL17A blockade

**Fig 7:**
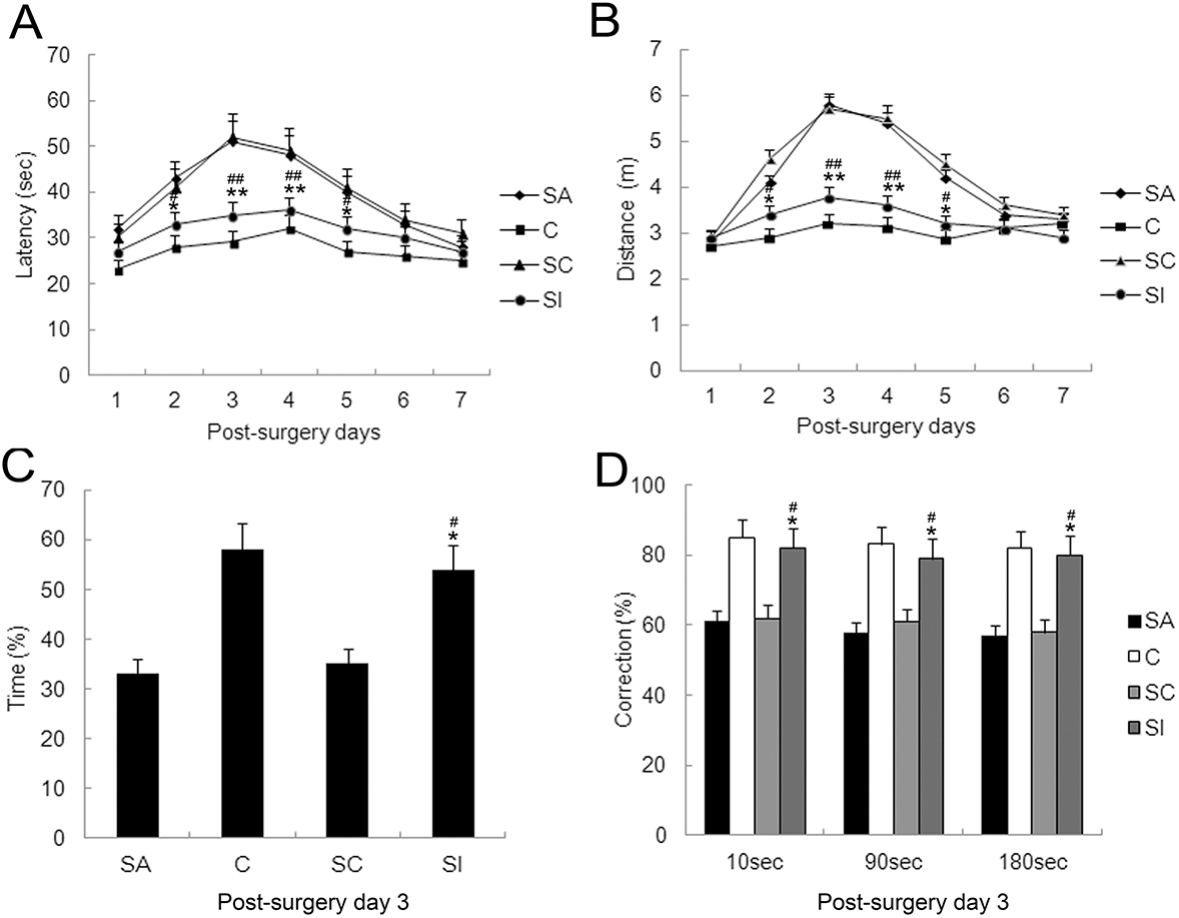
Surgery-induced cognitive impairment is prevented by concurrent administration of IL17A monoclonal antibody. Cognitive function was evaluated by Morris water maze (MWM) for 7 days after surgery and by a probe test on postoperative day 3 among mice treated with IL17A monoclonal antibody (SI) or IgG2a control (SC), surgery mice (SA) and control mice (C). (A) Swimming distance; (B) Swimming latency; (C) Time spent in the quadrant with the previously located hidden platform. (D) Spatial working memory was exmined by T maze. Data are represented as means ± SEM. *P<0.05; **P<0.01 vs. SC3 group and #*P*<0.05; ##*P*<0.01 vs. SA3 group (n = 8).

**Fig 8:**
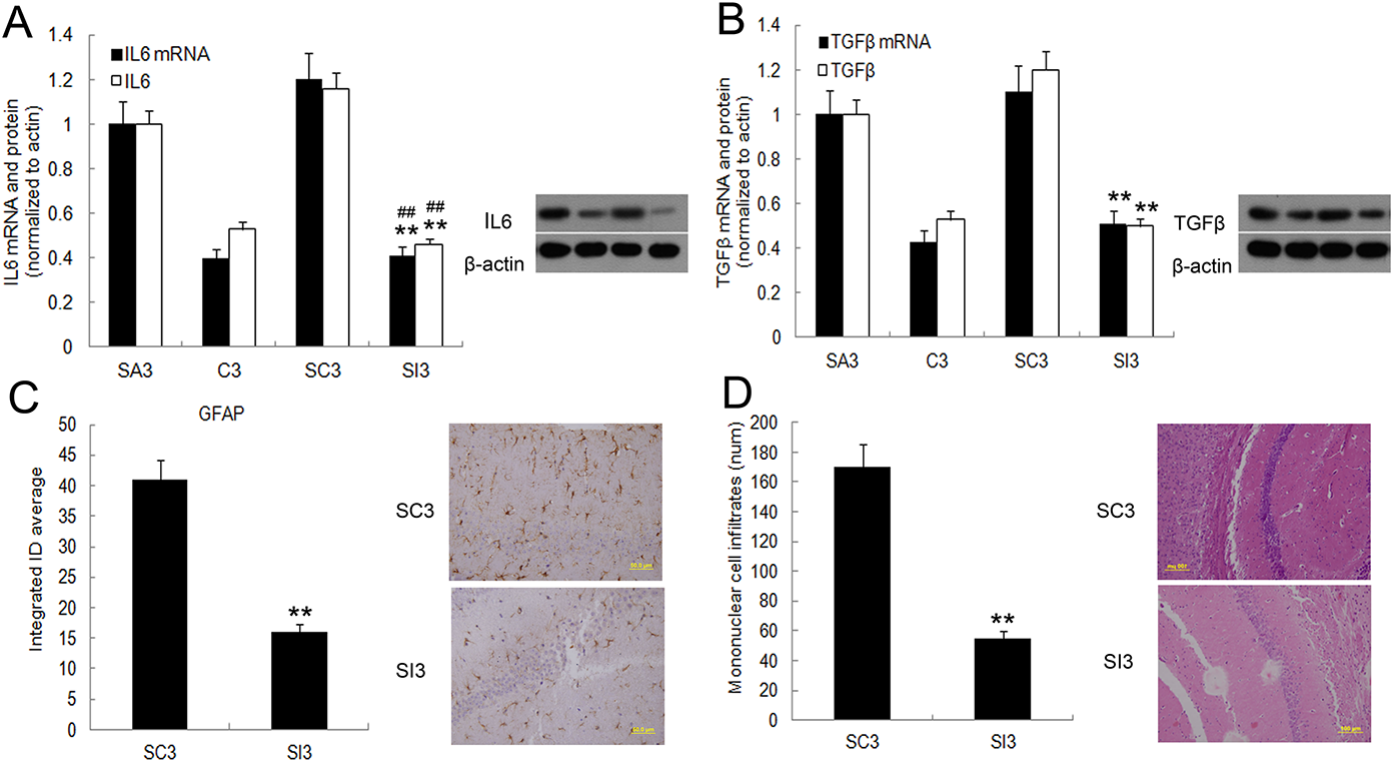
Mitigation of surgery-induced inflammation in mice by disabling or reducing IL17A signaling. Expression of IL-6 (A) and TGFβ (B) mRNA and protein in hippocampus was measured by qRT-PCR and western-blotting assay on postoperative day 3. (C) Expression of GFAP in hippocampus was measure by **i**mmunofluorescene assay (magnification ×400). (D) Histology of paraffin sections of hippocampus isolated from IgG2a control mice or mice with IL17A antibody ICV infusion **(**H&E magnification ×200**)** on day 3 after surgery. Data are represented as means ± SEM. *P<0.05; **P<0.01 vs. SC3 group and #*P*<0.05; ##*P*<0.01 vs. SA3 group. Groups were as follows: C3, control; SC3, IgG2a control; SA3, surgery; SI3, IL17A monoclonal antibody treated (postoperative day 3, n = 8).

**Fig 9:**
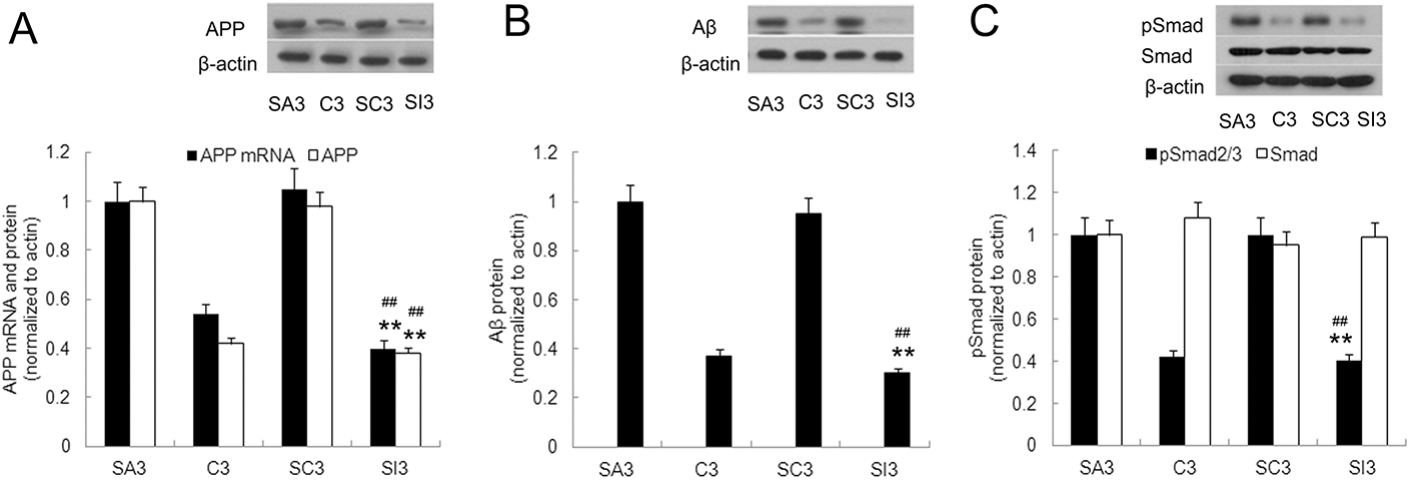
IL17A blockade in mice inhibits surgery-induced alterations in APP, Aβ_1–42_, and phospho-Smad2/3 levels. qRT-PCR and Western blots were used to measure mRNA and protein levels, respectively, of APP and Aβ_1–42_ in the hippocampus of all mice on postoperative day 3. Quantification of APP (A) and Aβ_1–42_ (B) mRNA and protein levels in surgery mice treated with IL17A monoclonal antibody (SI3) relative to levels in control IgG2a treated (SC3) and surgery mice (SA3). (C) phospho-Smad2/3 protein (pSmad) levels in surgery mice treated with IL17A monoclonal antibody (SI3), IgG2a control mice (SC3), and surgery mice (SA3). Data are represented as the mean ± SEM. *P<0.05; **P<0.01 vs. SC3 group and #*P*<0.05; ##*P*<0.01 vs. SA3 group (n = 8).

To explore the role of IL17A/ IL17 receptor (IL17R) signaling in surgery triggered hippocampus pathological injury, mice were administered either IL17A monoclonal antibody (group SI) or IgG2a (group SC) during surgery. In the MWM test, compared with group SC or group SA mice, group SI mice demonstrated a significant decrease in escape latency time and swim distance on postoperative day 3 (*P*<0.01, Fig 7A and 7B). Consistently, they spent significantly more time in the target quadrant than other quadrants than mice in group SC and SA (*P*<0.05, Fig 7C), suggesting that the SI mice were better in this spatial memory test. Moreover, spatial working memory was also tested by a T maze on postoperative day 3, for the 10 sec, 1 min or 3 min delay interval, SI group mice performed better than the SC or SA group (*P*<0.05, Fig 7D), but there is no difference between group SI and group C in either MWM or T maze test, suggesting that the SI mice were better in this spatial memory test. On postoperative day 3, hippocampal IL6 and TGF-β mRNA and protein levels were significantly less in group SI relative to group SC or SA (*P*<0.01, Fig 8A and 8B). The number of GFAP-positive cells was significantly less in group SI (*P*<0.01, Fig 8C). Histological analysis of hippocampus sections revealed that group SI mice developed mild inflammatory infiltration, whereas group SC mice showed prominent CNS pathology (*P*<0.01, Fig 8D). Lastly, the levels of APP, Aβ_1–42_, and Smad2/3 protein hyperphosphorylation on postoperative day 3 were significantly less in group SI relative to group SC or SA (*P*<0.01, Fig 9A–9C).

### IL17A blockade decreased astrocytic TGFβ, pSmad2/3, APP, and Aβ_1–42_ *in vitro*

**Fig 10:**
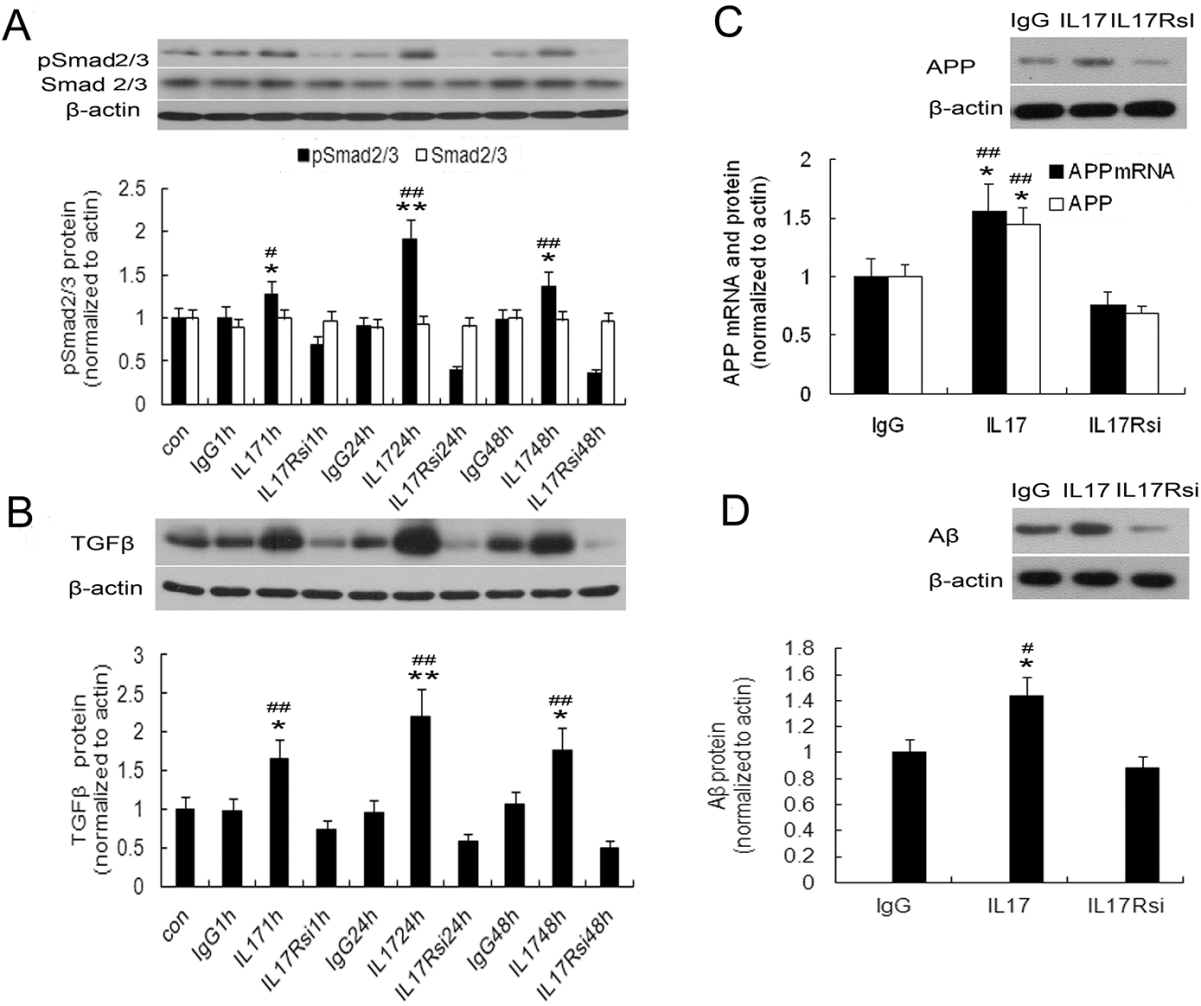
Blockade of IL17A inhibits TGFβ, phospho-Smad2/3 (pSmad2/3), APP, and Aβ_1–42_ production in mouse primary astrocyte cultures. Primary astrocytes were treated with IL17A (25 ng/ml, named IL17 group), rabbit anti-mouse IGg2a (25 ng/ml, named IgG group), or IL17A plus IL17R siRNA (100nM, named IL17Rsi group) for 1, 24, or 48 h. The protein was assessed with Western blotting specific for TGFβ (A), pSmad2/3 (B), APP (C) and Aβ_1–42_ (D). Data are represented as means ± SEM. *P<0.05; **P<0.01 vs. IgG2a group at the corresponding time point. #*P*<0.05; ##*P*<0.01 vs. IL17 Rsi group at the corresponding time point.

IL17R is expressed in most tissues examined to date, importantly in CNS glial cells [30, 31]. We hypothesized that astrocytes are a target of Th17 cells and IL17A in the CNS, and in order to investigate whether activated astrocytes decrease TGFβ and pSmad2/3 expression under IL17A-blocking conditions, IL17R small interfering RNA was used. Astrocytes were stimulated with medium (control), rat anti-mouse IgG2a, IL-17A, or IL17A plus IL-17R siRNA for up to 48 h, and expression of TGFβ and pSmad2/3 protein were analyzed by western blot. Levels of TGFβ and pSmad2/3 protein were elevated 1 h after the addition of IL17A, peaked at 24 h, and then decreased slightly at 48 h. When astrocytes were treated with both IL17A and IL17 R siRNA, the expression of TGFβ and pSmad2/3protein was significantly reduced at all time points (*P*<0.05 or 0.01, respectively; Fig 10A and 10B). IL17A had no significant effect on Smad2/3 protein expression. Taken together, these findings indicated that IL17A enhanced TGFβ and pSmad2/3 expression at the protein levels in astrocytes.

To determine whether the IL17A-stimulated astrocytes have elevated APP and Aβ_1–42_ expression, we prepared stimulated primary astrocyte cultures, as described above, and measured APP mRNA levels by quantitative RT-PCR. IL17A stimulation significantly upregulated astrocytic APP mRNA levels relative to control, and 24 h treatment with IL17 R siRNA attenuated this effect (*P*<0.05 or 0.01, respectively; Fig 10C and 10D). Similar findings were seen with APP and Aβ_1–42_ protein levels by western blot (*P*<0.05 or 0.01, respectively; Fig 10C and 10D). These data suggested that a significant proportion of the early IL17A-stimulated increases in APP and Aβ_1–42_ levels were blocked by IL17R siRNA in astrocytes.

### IL17A–enhanced astrocytic Aβ_1–42_ expression depending on the TGFβ/Smad pathway

**Fig 11:**
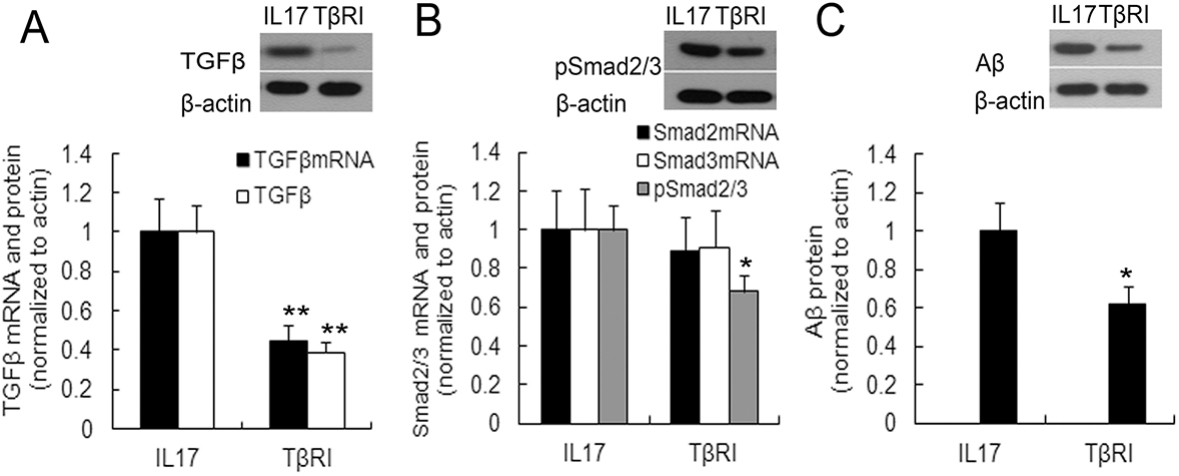
Inhibition of TGFβ/Smad signaling blocks the IL17A-stimulated increase in TGFβ, phospho-Smad2/3 (pSmad2/3), and Aβ_1–42_ levels in mouse primary astrocytes. Primary astrocytes were pre-treated for 30 min with TGFβ receptor inhibitor (TβRI, 5uM) then stimulated for 24 h with IL17A (25 ng/ml). Cells were harvested and analyzed for mRNA levels of TGFβ, pSmad2/3 by qRT-PCR, their proteins and Aβ_1–42_ protein was detected by Western blotting in IL17A (named IL17 group) alone or in combination with TβRI (named TβRI group) treatment. A. TGFβ, B. pSmad2/3, C. Aβ_1–42_. All data are the mean ± SEM. *P<0.05; **P<0.01 vs. IL17A group.

Previously, the TGFβ and Smad signaling pathway had been implicated in the regulation of Aβ_1–42_ production [32, 33]. Therefore, in this study, we sought to evaluate the role of TGFβ/Smad pathway in IL17A–enhanced astrocytic Aβ_1–42_ expression. Using specific pharmacological inhibitors for TGFβ/Smad, we found that inclusion of TGFβ/Smad inhibitor significantly suppressed IL17A induction of TGFβ, pSmad2/3, and Aβ_1–42_ expression in astroctyes after stimulation for 24h (*P*<0.05 or 0.01, respectively; Fig 11A–11C).

**Fig 12:**
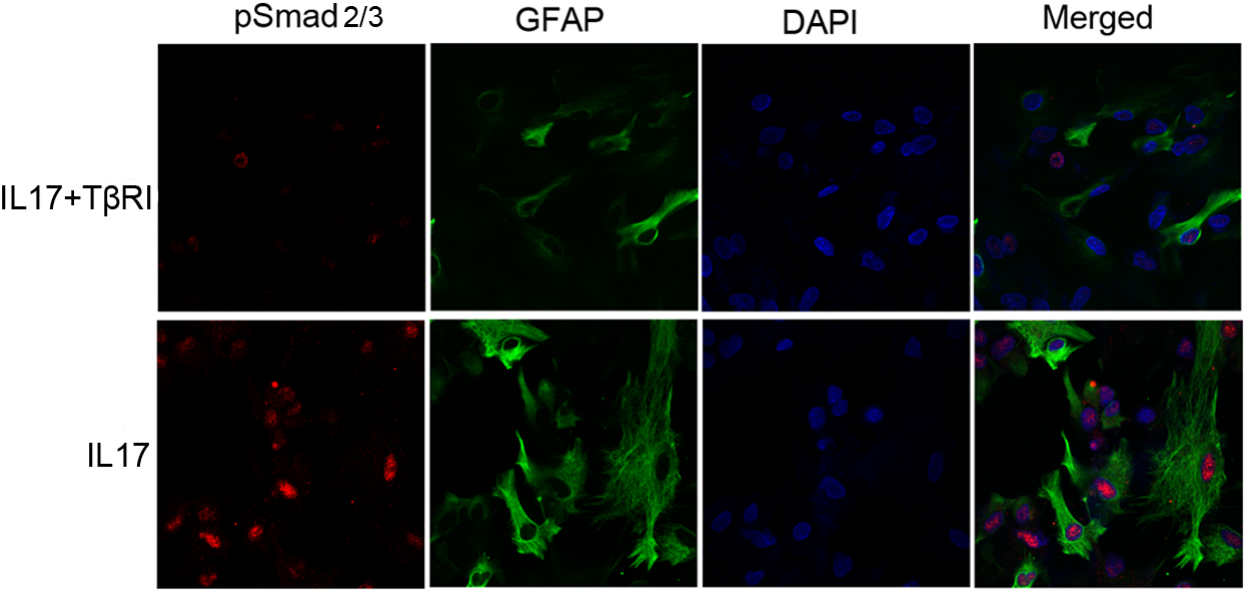
Inhibition of TGFβ signaling blocks IL17A-stimulated co-expression of phospho-Smad2/3 (pSmad) with GFAP in mouse primary astrocytes. The administration of TGFβ receptor inhibitor (TβRI) reduced IL17A-induced pSmad 2/3 co-expression with GFAP in astrocytes. Double immunofluorescence staining showed that relative to IL17A (25 ng/ml) alone, pSmad 2/3 (red) co-localization with GFAP (green) in astrocytes following IL17A in combination with TβRI (5 μM) treatment was reduced. Magnification × 600.

To further clarify the relationship between the TGFβ/Smad pathway and astrocytic activation under IL17A intervention, co-localization of pSmad2/3 with GFAP was investigated by double immunofluorescence labelling assay. Following IL17A treatment, pSmad 2/3 partially colocalized with GFAP in the astrocytes, whereas in IL17A plus TβR inhibitor (TβRI) treated cells, little GFAP and pSmad 2/3 were present in the cytoplasm and nucleus (Fig 12). These results indicated that in activated astrocytes, IL17A induced Aβ_1-42_expression by activating the astrocytic TGFβ/Smad signaling pathway.

## Discussion

POCD is one of the most common complications after cardiac and non-cardiac surgery and is independently associated with poor long-term outcome and even permanent dependence on social care systems in some patients [34, 35]. Clinical and basic research efforts focused on understanding the mechanisms and pathways involved are critical for improving management of POCD.

In the present study, we investigated the relationship between IL17A-triggered inflammatory response in the hippocampus and the development of POCD. After hepatectomy surgery under general anesthesia, mice exhibited memory impairment that was associated with activation of astrocytes and accumulation of Aβ_1–42_ in the hippocampus. Neither cognitive dysfunction nor Aβ_1–42_ accumulation was detected in mice subjected to anesthesia alone.

Surgery promote expression of proinflammatory cytokines, including IL1β, IL6 and TNFα in the hippocampus, and these changes may be involved with the development of POCD [5, 6, 7, 8]. In addition to confirming this finding, we identified a novel role for cytokine IL17A in POCD. Th17 cells produce IL17A and have been suggested to be the critical driver of autoimmune tissue inflammation, including collagen-induced arthritis, inflammatory bowel disease (IBD), and experimental autoimmune encephalomyelitis (EAE) [18, 20, 36, 37, 38]. IL17A signals become operative through a heteromeric receptor complex consisting of IL17RA and IL17RC, which are single-pass transmembrane proteins expressed by many cells [39, 40]. Until now, little was known regarding IL17A/IL17R axis involvement in POCD.

We found that hippocampal IL17A expression was significantly increased on postoperative day 1 and 3, with peak cognitive dysfunction on day 3, the day linked with activation of astrocytes. Meanwhile, on postoperative day 3, TGFβ expression was prominently enhanced, and Smad2/3 protein phosphorylation was increased in hippocampus. Target protein production of APP and Aβ_1–42_ was induced conspicuously on postoperative day 3, and Aβ_1–42_ levels remained elevated on postoperative day 7. When IL17A was blocked, surgery-triggered IL17A inflammation, which resulted in astrocyte activation, TGFβ/Smad activation, AD-like pathologic changes, and cognitive dysfunction, was abolished. These results suggested that in our surgical model, surgery triggered in hippocampus an immediate expression of IL17A, a gradual activation of astrocytes, and TGFβ/Smad molecular upregulation. Both our *in vivo* and *in vitro* studies are the first to show that cytokine IL17A-mediated POCD may share a common molecular mechanism with AD.

In AD, the inflammatory response is localized primarily within the vicinity of amyloid plaques. There is mounting evidence suggesting that inflammation in the central nervous system, particularly in the hippocampus, is mediated by the activation of glial cells and the production of inflammatory mediators (such as cytokines). These changes may contribute to the neuropathogenesis of AD [10, 41, 42, 43]. Indeed, the cognitive decline observed in this study was associated with the presence of the pathologic markers of AD, namely astrocyte activation and the accumulation of Aβ_1–42_. We postulated that POCD may be caused by the development of these postoperative pathologic changes induced by hippocampal IL17A. Several studies demonstrated that Aβ is directly toxic to cultured neurons, inducing cell degeneration and death that is apoptotic in nature [44, 45, 46]. It is this cell death that ultimately leads to the memory impairment and disorientation that is characteristic of AD and may also causally contribute to the POCD observed in this study.

Cytokine IL1β has a wide array of reported effects on cognitive dysfunction, ranging from enhancement of glutamate neurotoxicity [47] to the production of extracellular proteases, such as COX-2, PGE2 [48], and others. Likewise, IL17A may cause cellular damage and associated cognitive impairment, as seen in our present study, by promoting Aβ_1–42_ accumulation. The molecular mechanisms, however, by which IL17A increased the level of Aβ_1–42_ in astrocytes are unknown. Nevertheless, the observed increase in astrocytic TGFβ and pSmad levels following IL17A stimulation suggested that activated TGFβ and Smad signaling was at least partially responsible.

TGF-βs belong to a family of peptides that play important roles in intercellular signal transduction [49]. They transduce biological signals by activating a set of serine–threonine kinase receptors, then downstream activation of the Smad transcription factor cascade and the following regulation of transcription of TGFβ target genes [50]. To date, Smads are the only known substrates and signaling transducers of the activated TGFβ-receptors. However, it is unlikely the positive and negative changes in the gene expression induced by TGFβ signaling occur with Smad proteins alone. Smad-dependent transcriptional activation is probably modulated by the interaction of transcriptional co-activators or co-repressors [50]. In the astrocytes of AD, the TGFβ/Smad pathway plays a key role in the regulation of Aβ production [32, 33, 51, 52, 53]. To determine whether the TGFβ/Smad pathway was involved in the elevation of astrocytic Aβ_1–42_ levels in POCD, we pretreated primary astrocyte cultures with the TGFβ/Smad inhibitor followed by stimulation with IL17A. Not only were astrocytic Aβ_1–42_ levels reduced with TβRI, but there was a significant effect on astrocytic pSmad levels. Here, we demonstrated a direct link between TGFβ/Smad pathway activation and IL17A-triggered Aβ_1–42_ accumulation in mice after surgery.

In POCD, a positive feedback mechanism may operate to elevate and sustain astrocytic Aβ_1–42_ processing. Inflammatory cytokine IL17A stimulates: 1. astrocyte activation to produce more cytokines; 2. continued activation of TGFβ/Smad pathway; 3. increased levels of APP and secreted Aβ_1–42_ from astrocytes; and 4. further elevation of hippocampus Aβ_1–42_ levels, promoting production of more cytokines and Aβ_1–42_. There is some evidence in favor of the existence of this cycle. Aβ is capable of stimulating astrocytes to secrete inflammatory cytokines, and conversely, cytokine combinations, such as IL1, IL6, TNFα, and TGFβ, are capable of promoting astrocytic Aβ secretion. When working together, these events lead to a feedforward loop in the progression of the disease [54, 55, 56, 57, 58].

In summary, we have demonstrated that surgery triggers a transient neurocognitive decline in a mouse model that is associated with IL17A-induced astrocyte activation and consequent TGFβ/Smad pathway-dependent secretion of Aβ_1–42_ in the hippocampus. Strategies designed to attenuate this feed-forward mechanism may represent a means of preventing postoperative cognitive dysfunction.
